# Hereditary Colorectal Tumors: A Literature Review on MUTYH-Associated Polyposis

**DOI:** 10.1155/2017/8693182

**Published:** 2017-09-25

**Authors:** Micaella Kantor, Javier Sobrado, Sima Patel, Sara Eiseler, Christopher Ochner

**Affiliations:** ^1^Kendall Regional Medical Center, Miami, FL, USA; ^2^Larkin Community Hospital, South Miami, FL, USA

## Abstract

MAP (MUTYH-associated polyposis) is a syndrome, described in 2002, which is associated with colorectal adenomas, with enhanced colorectal carcinogenesis. This review synthesizes the available literature on MAP and outlines its pathogenesis, association with colorectal tumorigenesis, screening, treatment, and the subtle differences between it and its close cousins—FAP and AFAP. The preponderance of data is collected using MAP guidelines. However, although AFAP and MAP appear similar, potentially important distinctions exist, warranting targeted diagnostic criteria and treatment approaches. We suggest that it may be prudent to screen for MAP earlier than in current clinical practice, as it has been shown that sequence variants are associated with more severe disease, presenting with an earlier onset of colorectal cancer. Finally, we issue a call-to-action for much-needed further data to establish clear clinical and diagnostic criteria.

## 1. Introduction

Familial adenomatous polyposis (FAP) is an inherited disorder, which represents one of the most common gastrointestinal polyposis syndromes associated with colorectal tumorigenesis [1, 2]. A phenotypically milder and less understood form of FAP, attenuated familial adenomatous polyposis (AFAP), is associated with fewer adenomas and a later onset of colorectal cancer [1–3]. Both FAP and AFAP are associated with inherited mutations to the adenomatous polyposis coli (APC) gene [1–3]. Until 2002, a second gene, MUTYH, has been found in patients that present similar phenotypes, particularly to AFAP, in which no APC gene mutation was identified [2, 3]. Investigation revealed that this gene is involved in DNA base excision repair, and biallelic mutations of the MUTYH gene is related to the development of multiple adenomas, a syndrome known as MUTYH-associated polyposis or MAP [2, 3]. Formerly, MAP was diagnosed based on the presence of 20–99 adenomatous polyps; however, it has been discovered that these patients can present with sessile-serrated adenomas, hyperplastic polyps, and mixed polyps [3]. Unfortunately, many patients do not get diagnosed until they have already developed CRC.

## 2. Methods

### 2.1. Data Sources and Searches

We searched Ovid MEDLINE and PubMed databases to identify relevant articles indexed from 2002 to 2017. The search was conducted between February 1, 2017 and April 1, 2017, looking for publications that concern MYH, hMYH, MutY homolog, or MUTYH. Additional relevant articles from the reference lists of relevant studies were also used.

### 2.2. Study Selection

Eligible articles were published in English. Studies indexed prior to the discovery of MAP in 2002 were excluded. Individual case reports and case series were excluded. Given the relatively small number of studies published in this area, no further exclusion criteria were imposed.

### 2.3. Data Synthesis and Analysis

Heterogeneity within the available literature precluded the ability to conduct a formal quantitative synthesis of article findings. Therefore, we qualitatively synthesized the results and presented review data as a narrative summary [4].

## 3. Results and Discussion

### 3.1. FAP (Familial Adenomatous Polyposis) and MAP (MUTYH-Associated Polyposis)

Familial adenomatous polyposis (FAP) is a well-defined autosomal dominant disorder in which hundreds or thousands of synchronous colorectal adenomas develop early in life and inevitably lead to colorectal cancer (CRC) unless prophylactic colectomy is performed [1, 5]. Extracolonic manifestations are common and include polyps of the upper gastrointestinal tract, desmoid tumors, and osteomas [1–3]. The gene mutation is found in the APC (adenomatous polyposis coli) tumor suppressor gene on chromosome 5 [2]. Two less well-defined variants of FAP which cause benign colonic polyps with potential malignant transformation include attenuated familial adenomatous polyposis (AFAP) and MUTYH-associated polyposis (MAP) [3, 5, 6].

Attenuated familial adenomatous polyposis (AFAP), or oligopolyposis, is characterized by an older age of onset, usually between the ages of 40 to 70, with a fewer number of colorectal adenomas (<100) and a predilection to the proximal colon [1, 3, 5]. It is caused by mutations to the APC gene, but in the extreme distal or proximal portion [6]. Three specific regions identified are in the 5′ alternative splice region in exon 9 and the extreme 3′ part of the gene [1, 6]. In addition to these germline mutations, it has been proposed that modifier genes may be acting on the AFAP phenotype, influencing the amount of functional protein produced by the mutant allele, possibly causing this disease to be phenotypically and genetically heterogeneous [6].

MUTYH-associated polyposis (MAP) is the first known polyposis syndrome with a recessive mode of inheritance that usually appears in patient with an attenuated polyposis phenotype [1, 5]. Tubular adenomas tend to be the most frequent polyp reported; however, tubulovillous adenomas, hyperplastic polyps, and sessile-serrated adenomas have also been described [5, 7]. Biallelic mutations in the MUTYH gene, located at chromosome locus 1p34.3-p32.1, are responsible for the development of these polyps [5, 8]. Mutation carriers have polyps that range anywhere from ten to a few hundred, with a mean of 50 polyps; however, there have been cases of MAP presenting with over 500 polyps, as well as, patients with colorectal cancer (CRC) and no polyps [3, 5, 8]. Though less frequent, extracolonic manifestations similar to those seen in FAP have also been noted in some individuals [3]. Gastric and duodenal tumors are the most common manifestation; however, thyroid abnormalities and sebaceous skin tumors were commonly described [3, 8]. One study found the incidence of extraintestinal malignancies to be almost twice that of the general population, particularly for ovarian, bladder, skin, and breast cancer in MAP, compared to FAP [9, 10]. However, data is limited given the small population sample size studied in comparison to FAP.

MUTYH mutation analysis has become increasingly popular in patients with FAP-like and AFAP-like phenotypes in which no APC gene mutation is identified or when no clear evidence of vertical transmission is recognized [1, 11]. Using registries of polyposis, it has been documented that up to 40% of these cases were found to have biallelic MUTYH mutations [3, 8]. Due to the recessive mode of inheritance, MAP patients are often discovered late in the course of their disease, having already developed CRC as compared to FAP patients where the family genetics are known, yielding a predilection for early screening and diagnosis [10]. Sampson et al. [11] and Sieber et al. [12] reported the presence of CRC at presentation in approximately 50% of cases. Without treatment, colorectal cancer risk in patients with biallelic mutation is approximately 80% by the age of 70, with a mean age of diagnosis of 48 [3, 7, 13]. These implications support the importance of genetic diagnosis in affected families, leading to timely screening and early therapeutic intervention in hopes to prevent colorectal cancer.

### 3.2. MUTYH and BER

The mutY gene was first cloned in *Escherichia coli* in 1998 [8]. Recently, in 2002, the role of the MUTYH gene was discovered by Al Tassan et al. [14] in a Welsh family presenting with a recessive inheritance of multiple colorectal adenomas and CRC. These tumors were noted to have a significantly greater proportion of somatic mutations consisting of guanine-cytosine pair to thymine-adenine pair transversions in the APC gene [5, 8, 15]. These mutations are typically consistent with oxidative damage to DNA, therefore leading to the suspicion of a MUTYH protein deficiency [1, 8]. The MUTYH protein is a base excision repair (BER) glycosylase involved in the repair of oxidative damage, specifically oxidation of a guanine to 8-oxo-7,8-dihydro-2′-deoxyguanosine (8-oxoG), which then tends to mispair with adenine (instead of cytosine) [1, 8, 12]. Using a base-flipping mechanism, MUTYH recognizes and excises the adenine from the oxoG:A mismatch [1, 12]. Subsequently, DNA polymerases and OGG1, another BER glycosylase, restore the oxoG:C pair and replace the oxidized guanine with a guanine, respectively [1, 8, 12, 15]. Therefore, during oxidative stress, both MUTYH (including MTH1 in the C terminal domain, which recognizes 8-oxoG) and OOG1 act synergistically to maintain genome stability and prevent tumorigenesis [1, 8]. Before the discovery of MAP, no inherited deficiencies of BER have been linked with disease [1].

A dysfunctional MUTYH protein generates somatic G>T transversions in multiple genes, including APC and K-ras [3, 5, 8]. Its behavior resembles that of a pleiotropic gene, with a variable phenotypical presentation including AFAP-like syndromes, as seen in the Welsh family [2, 14], serrated adenomas, hyperplastic polyps, and sporadic colorectal cancers [5, 7].

Genetic pathways of CRC development in MAP are currently being extensively studied, particularly histologic and molecular genetic findings on tumor tissue. Analysis of multiple adenomas revealed not only APC mutations, involving the initiation of G to T transversions, but also K-ras mutations, specifically a missense mutation in codon 12 (c.34G>T) [8, 16]. APC mutations were found in 14–83% and K-ras mutations in about 64% of CRCs [8, 16]. Further investigation revealed that the G>T transversions found in these mutated genes are responsible for early tumor development, suggesting MUTYH may have a role in early carcinogenesis [4, 8]. Nielson et al. [8] found that MAP CRCs, as well as those with Lynch syndrome, tend to have better overall survival than sporadic CRC. Though microsatellite instability (MSI) is not characteristic, MAP CRCs show histological overlap with MSI-high and Lynch syndrome CRCs in that they tend to present with proximal lesions, have a high rate of mucinous histotype, and increased presence of tumor-infiltrating lymphocytes (TILs) [8].

### 3.3. Identification and Characterization of MAP

The diagnosis of MAP has primarily been suspected in individuals based on the presence of 20–99 adenomatous colonic polyps, commonly tubular adenomas [7]. Until recently, it has been discovered that MAP can also present unconventionally with sessile-serrated adenomas (SSAs), hyperplastic polyps (HPs), and mixed polyps (hyperplastic and adenomatous), differentiating MAP from other polyposis syndromes [5, 7, 8]. Boparai et al. [5] found that 47% of patients with MAP had HPs/SSAs along with adenomas. Forty-one percent of the adenomas had APC gene mutations, while 23% of them had K-ras mutations [5]. However, when they analyzed HPs and SSAs, 70% had K-ras mutations, and none had APC mutations [5]. They also further noted that 18% of the patients met the criteria for serrated polyposis syndrome, a recently recognized condition requiring at least 5 HPs proximal to the sigmoid, 2 of which are greater than 10 mm, or of more than 20 HPs throughout the colon [5].

To date, over 300 unique sequence variants have been reported for the MUTYH gene, being predominantly missense mutations [8, 9]. While testing for oxidative repair genes for germline changes, Al Tassan et al. [14] found two missense variants in the MUTYH gene, Y179C and G396D (previously referred to as Y165C and G382D, resp.), in the affected family members. Today, p.Y179C and p.G396D are the two most common mutations, representing 80% of MAP cases in the Caucasian population [3, 7, 8, 12, 14]. Since these mutations were not found in the Asian population, nor in Jewish persons of European origin, ethnic differentiation and founder mutations are also characteristic [7].

Using functionality assays to further analyze sequence variants, it was found that patients with p.Y179C mutation had significantly reduced MUTYH glycosylase activity in comparison to those with pG396D [8, 9, 17]. Therefore, phenotypically, patients with the homozygous p.Y179C mutation presented with more severe disease, when compared to those with biallelic p.G396D or those with compound heterozygous p.G396D/p.Y179C [7, 17]. Furthermore, Nielson et al. [8] demonstrated that patients with homozygous p.G396D or compound heterozygous p.G396D/p.Y179C mutation presented with the diagnosis of MAP later and had a lower hazard for developing CRC, with the mean age of CRC diagnosis of 58 years and 52 years, respectively [8]. Those with homozygous p.Y179C presented with CRC at an average of 46 years [3, 8]. These findings should be considered when developing personalized screening and therapeutic strategies, given the significant aggressive nature of certain sequence variants.

### 3.4. Screening and Management of MAP

When the diagnosis of a polyposis syndrome is established and pedigree suggests autosomal dominant inheritance, screening for APC mutation via molecular gene testing should be the first step. Because mutations are found in various regions of the gene, analysis of the entire APC should be performed [3]. If a family with AFAP-like syndrome implies recessive inheritance (i.e., one or more cases in one generation), one should screen for MUTYH mutations. One study found that the combination of >15 synchronous adenomas and the development of CRC in patients younger than 50 years of age generated a sensitivity of 75% and a specificity of 93.8% for the detection of biallelic MUTYH mutations [17]. In the Western population, the majority will present with at least one mutation in Y179C and G396D; however, the presence of population-specific mutations should be considered in those with different ethnic backgrounds; therefore, it is recommended to screen the entire MUTYH gene [1, 8].

Differentiating MAP from other polyposis syndromes is particularly important because the siblings are those at greater risk (25%) for developing the disease, as opposed to the offspring, due to autosomal recessive transmission [2, 8]. Since 1-2% of the populations is found to have monoallelic MUTYH mutations [8], testing the spouse of those found to have MAP can be considered, since this may pose a risk to future offspring.

Currently, CRC screening and treatment for MAP follows the same principles as for AFAP. Patients with clinical suspicion of an adenomatous polyposis syndrome should undergo screening for CRC by annual colonoscopy [1, 3]. Flexible sigmoidoscopy, which can be done in classic FAP, is not recommended in AFAP nor MAP given their predilection for the proximal colon [3, 6, 8, 18]. Onset of examination can be delayed until the late teens or early 20s and should include endoscopy [10, 18]. Upper endoscopy should be performed every three to five years at age 25–35, depending on disease severity [3, 9, 18]. Studies have found the average of cancer diagnosis in AFAP to be 58 years (range, 29–81) and MAP to be 46 years [3, 8]. Therefore, although speculative supportive evidence is currently lacking, we suggest that it may be prudent for clinicians to screen some patients earlier, particularly those with risk of more severe disease (e.g., those with homozygous p.Y179C).

Documented or suspected cancer is an absolute indication for immediate colorectal surgery [1, 3]. Relative indications include multiple adenomas > 6 mm, presence of high-grade dysplasia in an adenoma, an acute increase in adenoma number, and when multiple small polyps are present making adequate colon surveillance difficult [3, 6].

## 4. Conclusion

Most homozygous MUTYH patients will develop 10 to 500 polyps, but there have been patients described with CRC with no polyps, or >500 [3, 5, 8]. It has been shown that HPs and SSAs, along with the presence of adenomas, are casually related to this mutation, and therefore can aid possible diagnosis [5, 7]. MAP etiology should be suspected in CRCs that present at a young age, the presence of polyps, and with a recessive inheritance pattern. CRCs in MAP tend to have better survival when compared to sporadic CRCs possibly because of the high number of TILS found, indicating active immune response [8].

Although many questions are yet to be answered when it comes to diagnosis, counseling, and screening/therapeutic strategies for patient with MUTYH mutations, it has been receiving increased attention in those patients presenting with polyposis coli and colorectal cancers. MUTYH germline molecular genetic testing is currently the standard for diagnosis, considered positive when biallelic MUTYH pathogenic variants are established in a proband. Though a positive test provides the diagnosis of a syndrome, interpretation of a negative genetic test can present a diagnostic challenge, given the chance of false negatives since many mutations have not yet been identified, or due to numerous sequence variations.

At this juncture, clinical assessment involving the age of onset of the first adenoma, number of adenomas, and personal or family history of CRC are crucial in selecting patients for genetic testing. Somatic KRAS analysis on tumor tissue has been suggested as a prescreening test to further select patients with CRC for germline genetic testing, particularly in those with atypical presentation (CRCs with no or few polyps) [8]. We suggest that it may be prudent to screen for MAP earlier and in shorter intervals in those with specific gene sequence variants, as these imply more severe disease. Without timely surveillance, CRC in MAP confers a lifetime risk of almost 100%. Therefore, there is an urgent need for the establishment of clear clinical and diagnostic criteria so that clinicians may intervene earlier and potentially decrease morbidity and mortality in patients with MAP.
